# Pathology

**Published:** 1907-03

**Authors:** I. Walker Hall


### PATHOLOGY.

The Pathology of to-day falls easily into three great divisions —morbid anatomy, bacteriology, and chemical pathology. The morbid anatomist is the one faithful to old traditions, belonging to the cave-dwellers who see things dimly while lingering over the minutiæ of dead bones and tissues; the bacteriologist is the adventurer who leaves old memories behind and soars into the unknown ether on the gossamer wings of Ehrlichism; the pathological chemist is the pacemaker who, while unravelling the constitution of substances, provides the necessary armament for the morbid anatomist and the chemical details for the bacteriologist. May a fourth class be added—the clinical pathologist, that *rara avis* who sometimes exchanges the coat of the laboratory for the plumes of the consultant!

*Convulsions.*—McIlraith[2] has just concluded an examination of 250 cases of convulsions occurring in infancy and childhood. He finds that predisposing causes play a more important part than do the exciting causes. The chief predisposing causes are "an inherited neurotic taint," and rickets. The chief exciting causes are those connected with gastro-intestinal disorders. Convulsions are by no means as frequent as generally supposed

[1] *Ibid.*, p. 732. [2] *Medical Chronicle*, 1906–7.

at the onset of acute fevers, such as measles and pneumonia. They are more common at the onset of pneumonia than at the onset of measles.

In children suffering from convulsions there is frequently a history of ill-health or disease in the parents, especially in the mother during pregnancy, and this acts by lowering the vitality of the child and rendering it more liable to disease.

Only in a very small proportion of cases convulsions can be ascribed to injury at birth, or to organic disease of the brain. In early life convulsions may be the first sign of epilepsy, or may give rise to that condition in later life, but distinct epilepsy is more likely to supervene when there is no obvious cause for the early convulsive attacks.

Dentition is rarely a cause of convulsions, and only when some predisposing cause exists. *Cherchez l'intestin* appears to be a golden rule for infantile convulsions.

*Tumours.*—Among the countless hypotheses which, like the many airships, rise only to fall again, the relation of trauma to the site and ætiology of sarcomatous changes has been strongly advocated by a number of recent writers. "*Ohne trauma, ohne sarcoma*" is the utterance of one enthusiast. Many clinicians could cite cases which appear to support this contention. The point of view depends on the interpretation of the word "trauma." Hardly a day passes without the occurrence of knocks—physical or mental—and imagination frequently aids the note-taker. Nevertheless, though the idea will rapidly receive its quietus from writers sufficiently vigorous to talk it down, yet there is much in it. Incidentally, Sternberg has just published his work on *Trauma in Internal Diseases*, and we are again reminded that pathological changes are not concluded when the scar tissue covers over the external wound. Whether it be lung, liver, kidney, or bones, the seat of once damaged tissues must always be regarded with suspicion. There is ever the possibility of growth, or malnutrition, to think of in connection with an injury.

*Fæces.*[1]—The clinician looks always at the fæces—preferably through a telescope or the glass wall of a closed vessel—and his assiduity is sometimes rewarded by the observation of unusual appearances. Diagnosis may frequently be assisted by more systematic investigations. Here are a few points culled from several recent papers on the subject.

The insufficiency of the functions of the stomach are best gauged by the amount of connective tissue in the fæces.

The extent of the deficiency of pancreatic digestion may be determined by the amount of nuclein in the fæces. This should be supplemented by estimations of the fat, but in itself is a very useful sign.

Pancreon is a preparation of pancreas tissue. If steatorrhœa

[1] *Centralbl. f. Allge. Path.*, 1906.

or azotorrhœa disappear after its administration, then it is probable that pancreatic functions are suspended.

*Tuberculosis.*—Many recent works[1] have been directed to the paths of the infection in tuberculosis. It is declared that the chief avenues are via the bronchi by inhalation, and the pharynx by swallowing. The tubercle bacilli readily passes through mucous membranes without leaving any evidence of its passage.

Schlossman affirms that tuberculosis is, in the greater number of cases, a disease of childhood. Infection may occur by means of tuberculous food, but this is a rare occurrence compared with that of infection from man. The child inhales air containing tubercle bacilli, or touches infected material with the finger, and, placing the latter in the mouth, is thereby contaminated. As many as fifty per cent. of adults show signs of tuberculosis before they are fully developed. The mortality amongst children is very great, and the fight against infection of children through parents and relations is a field which offers a prospect of great success in not only reducing the child mortality from tuberculosis, but also in reducing the numbers of those adults whose latent, oft-times unsuspected tuberculous conditions are a source of danger to the community, as well as a death-warrant to themselves.

I. WALKER HALL.

---

### Reviews of Books.

[1] "Reports of Hague Congress for Tuberculosis," 1906, in *Centralbl. f. Allge. Path.*, 1906.

